# Detection of Phosphorylated Alpha-Synuclein in the Muscularis Propria of the Gastrointestinal Tract Is a Sensitive Predictor for Parkinson's Disease

**DOI:** 10.1155/2020/4687530

**Published:** 2020-09-23

**Authors:** Goichi Beck, Yumiko Hori, Yoshito Hayashi, Eiichi Morii, Tetsuo Takehara, Hideki Mochizuki

**Affiliations:** ^1^Department of Neurology, Osaka University Graduate School of Medicine, 2-2 Yamadaoka, Suita, Osaka 565-0871, Japan; ^2^Department of Pathology, Osaka University Graduate School of Medicine, 2-2 Yamadaoka, Suita, Osaka 565-0871, Japan; ^3^Department of Gastroenterology and Hepatology, Osaka University Medical School, 2-2 Yamadaoka, Suita, Osaka 565-0871, Japan

## Abstract

**Background:**

Parkinson's disease (PD) is a neurodegenerative disorder characterized by motor and nonmotor impairments, including constipation. Lewy bodies and neurites, the pathological hallmarks of PD, are found in the enteric nervous system (ENS) as well as the central nervous system. Constipation is a well-documented premotor symptom in PD, and recent reports have demonstrated Lewy pathology in gastrointestinal (GI) tissues of PD patients prior to the onset of motor symptoms.

**Objective:**

In the present study, we assessed Lewy pathology in the GI tracts of seven PD patients who had undergone a gastrectomy, gastric polypectomy, or colonic polypectomy prior to the onset of motor symptoms in order to assess whether the presence of pathological *α*Syn in the ENS could be a predictor for PD.

**Methods:**

GI tissue samples were collected from control patients and patients with premotor PD. Immunohistochemistry was performed using primary antibodies against *α*-synuclein (*α*Syn) and phosphorylated *α*Syn (p*α*Syn), after which Lewy pathology in each sample was assessed.

**Results:**

In all control and premotor PD patients, accumulation of *α*Syn was observed in the myenteric plexus in both the stomach and colon. In 82% (18/22) of control patients, mild-to-moderate accumulation of *α*Syn was observed in the submucosal plexus. However, there was no deposition of p*α*Syn in the ENS of control patients. In patients with premotor PD, abundant accumulation of *α*Syn was observed in the myenteric plexus, similar to control patients. On the other hand, p*α*Syn-positive aggregates were also observed in the nerve fibers in the muscularis propria in all examined patients with premotor PD (100%, 3/3), while the deposition of p*α*Syn in the submucosal plexus was only observed in one patient (14%, 1/7).

**Conclusion:**

Our results suggest that the detection of p*α*Syn, but not *α*Syn, especially in the muscularis propria of GI tracts, could be a sensitive prodromal biomarker for PD.

## 1. Introduction

Parkinson's disease (PD), one of the most prevalent neurodegenerative disorders, is characterized by the progressive degeneration not only of the dopaminergic nigrostriatal system, which is responsible for the core motor symptoms including tremor at rest, bradykinesia, and rigidity [1, 2], but also by the involvement of many other neuronal systems and organs affected by different nonmotor deficiencies, including olfactory dysfunction, cardiac involvement, and REM (rapid eye movement) sleep behavior disorder [3]. Moreover, PD patients often experience symptoms that span the entire alimentary tract including dysphagia, delayed gastric emptying, constipation, and defecatory dysfunction [4, 5].

The postmortem diagnosis of PD requires not only evidence of dopaminergic cell loss in the substantia nigra but also Lewy pathology, or the widespread occurrence of intracytoplasmic depositions of phosphorylated *α*-synuclein (*α*Syn), the major protein marker and biological hallmark of PD and other synucleinopathies [6]. *α*Syn can undergo several posttranscriptional modifications, including nitration [7], ubiquitination [8], and SUMOylation [9]. However, more than 90% of the *α*Syn that accumulates in PD brains is phosphorylated at Ser129, and immunohistochemistry using an anti-phosphorylated *α*Syn (p*α*Syn) antibody is the strongest tool to detect Lewy pathology [6, 10].

Besides the central nervous system, Lewy pathology is observed within the sympathetic and parasympathetic ganglia [11], adrenal glands [12], enteric nervous system (ENS) [13–16], and cutaneous nerves [17]. The clinical diagnosis of PD depends on the appearance of cardinal motor symptoms, which are signs that do not appear before the loss of an estimated 70–80% of striatal dopamine [2, 18]. It is important to diagnose the disease earlier in order to maximally benefit from the numerous therapies targeting this disease.

PD patients often experience prodromal symptoms such as olfactory dysfunction, constipation, fatigue, and behavioral and mood changes [3, 19, 20]. It is now generally accepted that a variety of nonmotor features of PD are part of the evolving disease spectrum and commonly occur prior to the evaluation of the defining motor signs [3, 20]. Moreover, postmortem studies of incidental Lewy body disease suggest that *α*Syn pathology may begin in tissues of the gastrointestinal (GI) tract, salivary gland, and olfactory system [14, 15, 21, 22]. These studies lead to the proposal that, in a large proportion of PD cases, the substantia nigra is involved only after the olfactory system and lower brainstem regions [3].

In 2012, Shannon et al. demonstrated the accumulation of *α*Syn in the colonic mucosa and submucosa in PD patients before the development of characteristic motor symptoms [23], and more recently, Stokholm et al. have demonstrated the presence of Lewy pathology (aggregated p*α*Syn) in GI tissues of premotor PD patients [24], suggesting that deposition of *α*Syn in the ENS could be a useful predictor for PD. However, these studies have not considered the difference in Lewy pathology between the submucosa and muscularis propria. The objective of this study is to discover a more sensitive biomarker for premotor PD patients. To this end, we focused on Lewy pathology in the muscularis propria of GI tracts and compared it with that in the submucosa.

## 2. Materials and Methods

### 2.1. Subjects

Seven PD patients aged 53–79 years were recruited from the PD database of the Department of Neurology of Osaka University Hospital. A diagnosis was made according to the United Kingdom Parkinson Disease Research Society Brain Bank criteria [1]. The criteria for their recruitment were as follows: (1) a distal gastrectomy, gastric polypectomy, or colonic polypectomy was performed at the Osaka University Hospital before they exhibited any motor symptoms and (2) tissue samples taken by surgery were available. The clinical profiles of these patients are summarized in Table 1. Patient P1 developed dysphagia as the initial symptom of PD three months after the distal gastrectomy. Three patients (P1, P2, and P7) had constipation at the time of operation.

Control cases were selected randomly. Control samples were taken from four autopsy subjects, four patients with advanced gastric cancer, four patients with colon cancer, five patients with early gastric cancer or gastric polyps, and five patients with colonic polyps without a history of neurological or psychiatric diseases, respectively, from Osaka University Hospital. The eighteen patients with advanced gastric cancer, early gastric cancer, gastric polyps, colon cancer, or colon polyps (patients C5–C22) showed no neurological signs for at least six years after the operation. The profiles of the control patients are summarized in Table 2.

This study was approved by the Ethics Committee of Osaka University Hospital (no. 12148) and conducted in accordance with the Declaration of Helsinki (1964). The experiment was conducted with the human subjects' understanding and consent.

### 2.2. Immunohistochemistry

Tissue samples were fixed in 10% formalin and then dehydrated and embedded in paraffin blocks, and five-micrometer-thick paraffin serial sections were prepared. Consecutive slices were considered as the same site in each sample. Deparaffinized sections were incubated for 30 min with 0.3% H_2_O_2_ to quench any endogenous peroxidase activity, after which they were washed with PBS. The primary antibodies used were a rabbit polyclonal antibody against *α*Syn (Sigma-Aldrich (S3062), St. Louis, MO), a mouse monoclonal antibody against p*α*Syn (Wako Pure Chemical Corp. (pSyn #64), Osaka), and a rabbit polyclonal antibody against protein gene product 9.5 (PGP9.5, neuronal marker, Abcam (ab10404), Cambridge, UK). Autoclave treatment was performed for 15 min before incubation with all the antibodies. Goat anti-rabbit and anti-mouse immunoglobulins conjugated to peroxidase-labeled dextran polymer (Dako Envision+, Dako Corp., Carpinteria, CA) were used as secondary antibodies. Reaction products were visualized with 3,3′-diaminobenzidine tetrahydrochloride (Vector Laboratories, Burlingame, CA), and hematoxylin was used to counterstain the cell nuclei.

The staining pattern of *α*Syn was evaluated according to the following four-grade system: (1) strong, with more than half of the myenteric and/or submucosal plexus in each section and intramuscular nerve fibers strongly immunopositive for *α*Syn; (2) moderate, with an intermediate level between strong and weak immunoreactivity, with weakly positive intramuscular nerve fibers; (3) weak, with only a few plexuses in each section positive for *α*Syn and intramuscular nerve fibers negative; and (4) absent, with no immunostaining for *α*Syn. The expression level of *α*Syn was scored according to the following system: strong = score 3, moderate = score 2, weak = score 1, and absent = score 0. Three sections from each patient were examined by two specialists of pathology. The scores between the two groups were statistically compared by *t*-test, and statistical significance was determined at *p* < 0.05. The intraclass correlation coefficients (ICC) were calculated using Bell Curve for Excel (Social Survey Research Information Co., Ltd., Tokyo, Japan). The staining pattern of p*α*Syn was divided into two groups according to whether p*α*Syn-positive aggregates were detected (positive) or not (negative).

## 3. Results

The results of immunohistochemistry are summarized in Table 3.

**Figure 1:**
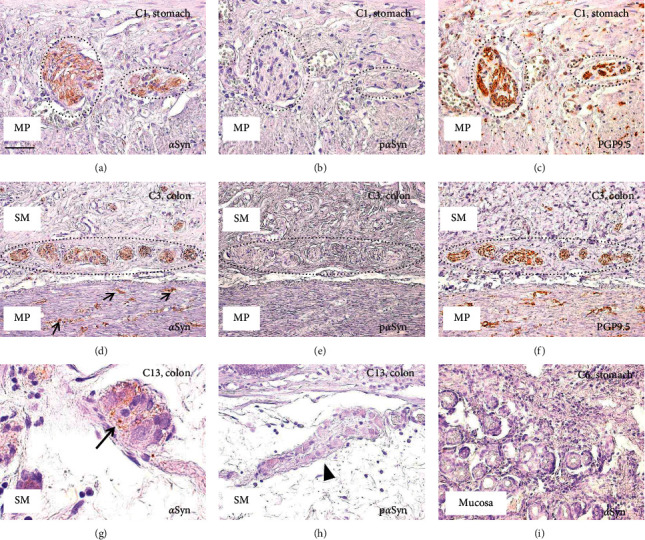
Immunohistochemical analyses of control patients. Immunohistochemistry for *α*-synuclein (*α*Syn) (a, d, g, i), phosphorylated *α*-synuclein (p*α*Syn) (b, e, h), and PGP9.5 (c, f). (a–c) Sections of the stomach of patient C1; (d–f) sections of the colon of patient C3; (g, h) sections of the colon of patient C13; (i) sections of the stomach of patient C6. (a–f) Serial sections. Black circles indicate the myenteric plexus (a–c) and submucosal plexus (d–f). MP, muscularis propria; SM, submucosa. Accumulations of *α*Syn are observed in gastric myenteric plexus (a), intramuscular nerve fibers (small arrows in (d)), and colonic submucosal plexus (arrow in (g)), but not observed in the mucosa (i). Depositions of p*α*Syn are visible neither in the gastric myenteric plexus (b) nor in the colonic submucosal plexus ((e) and arrowhead in (h)). Components of the enteric nervous systems are visualized with PGP9.5 (c, f). Scale bar: 50 *μ*m (a–c, h, i), 100 *μ*m (d–f), and 20 *μ*m (g).

In all control patients whose muscularis propria could be analyzed (patients C1–C12), *α*Syn immunoreactivity was detected in the myenteric plexus in both the stomach (Figure 1(a)) and colon. Six samples from six patients showed strong *α*Syn immunoreactivity, eight samples showed moderate *α*Syn immunoreactivity, and one showed weak *α*Syn immunoreactivity, respectively, in the muscularis propria (Table 3). In eleven cases, the accumulation of *α*Syn was observed in the intramuscular nerve fibers (Figure 1(d)) in addition to the myenteric plexus. However, phosphorylated *α*Syn (p*α*Syn) immunoreactivity was not detected in the muscularis propria in any of the control patients (Figures 1(b) and 1(e)). The components of the ENS were confirmed by immunohistochemical staining with PGP9.5 in the serial sections (Figures 1(c) and 1(f)). In the submucosal plexus, the accumulation of *α*Syn was observed in sixteen out of twenty-two control patients (Figures 1(d) and 1(g)). On the other hand, immunoreactivity for *α*Syn was not visible in the mucosa (Figure 1(i)) and depositions of p*α*Syn were not detected in either the submucosa (Figures 1(e) and 1(h)) or mucosa of any control patients.

**Figure 2:**
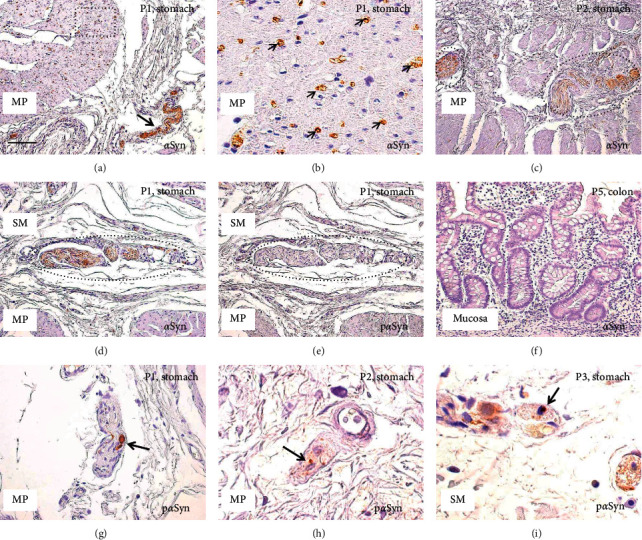
Immunohistochemical analyses of patients with premotor PD. Immunohistochemistry for *α*-synuclein (*α*Syn) (a–d, f), and phosphorylated *α*-synuclein (p*α*Syn) (e, g–i). (a, b, d, e, g) Sections of the stomach of patient P1; (c, h) sections of the stomach of patient P2; (f) section of the colon of patient P5; (i) sections of the stomach of patient P3. (b) High magnification view of the dotted square in (a, d, e) are serial sections. Black circles indicate myenteric plexus (c) and submucosal plexus (d, e). MP, muscularis propria; SM, submucosa. Accumulations of *α*Syn are observed in the nerve bundle in the MP (arrow in (a)), intramuscular nerve fibers ((b), small arrows), myenteric plexus (c), and submucosal plexus (d), but not in the mucosa (f). p*α*Syn-positive aggregates are observed in the nerve bundles in the MP (arrows in (g, h)) and submucosal plexus (arrow in (i)). In patient P1, the submucosal plexus with high accumulation of *α*Syn (d) shows no staining for p*α*Syn (e). Scale bar: 100 *μ*m (a, c), 50 *μ*m (d–g), and 20 *μ*m (b, h, i).

**Figure 3:**
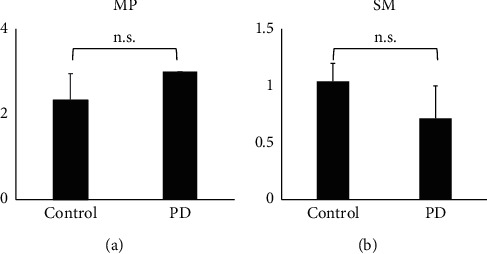
Scores of *α*Syn expression. In both the (a) muscularis propria (MP) and (b) submucosa (SM), there was no statistically significant difference in *α*Syn-expression scores between the control and PD groups. Data are shown as mean ± SEM. n.s, not significant.

In patients with premotor PD, we could analyze the muscularis propria in three cases (P1–P3) with gastric cancer. In all three cases, strong *α*Syn immunoreactivity was observed in the myenteric plexus (Figures 2(a) and 2(c)) and the intramuscular nerve fibers (Figure 2(b)), which was similar to some control subjects (Figures 1(a) and 1(d)). In the submucosa, weak-to-moderate *α*Syn immunoreactivity was visible in four out of seven (57%) PD patients (P1–P4) (Figure 2(d)). As shown in Figure 3, there was no significant difference in *α*Syn-expression scores between control and PD patients in both the muscularis propria (*p* > 0.05; ICC = 0.91) and submucosa (*p* > 0.05; ICC = 0.87). Most of those submucosal plexuses showed no p*α*Syn immunoreactivity (Figure 2(e)). Depositions of p*α*Syn-positive aggregates were detected in the nerve fibers in the muscularis propria in all three cases (3/3, 100%, P1–P3) (Figures 2(g) and 2(h)), while p*α*Syn-positive aggregates in the submucosal plexus were found in only one (P3) (Figure 2(i)) out of seven patients (1/7, 14%). Similar to the control patients (Figure 1(i)), *α*Syn immunoreactivity was not detectable in the mucosa in any of the premotor PD patients (Figure 2(f)).

## 4. Discussion

In this study, we demonstrated the accumulation of pathological *α*Syn in the muscularis propria of GI tracts in all examined premotor PD patients, although it was observed in the submucosa of only 14% of premotor PD cases. The accumulation of *α*Syn, which exists ubiquitously in the nervous system [25], was visible in the GI muscularis propria in both control and premotor PD groups. Our results suggest that the deposition of p*α*Syn, but not nonphosphorylated *α*Syn, in the muscularis propria could be a more sensitive and useful biomarker for premotor PD. It should be noted that we may be overlooking Lewy pathology by examining only samples taken by biopsy or endoscopic mucosal resection, since the muscularis propria is not included in these samples.

In control subjects, the accumulation of *α*Syn was observed, at one level or another, more prominently in the muscularis propria than in the submucosa. In addition, in patients with premotor PD, the most prominent accumulation of *α*Syn was observed in the muscularis propria, which was indistinguishable from the control group. These results suggest that the accumulation of *α*Syn, probably with aging, might initially occur in the muscularis propria and then spread out into the submucosa. Another possibility is that some unknown mechanisms in the submucosa might prevent the formation of pathological *α*Syn. Our results demonstrated that immunohistochemical analyses with *α*Syn antibodies may provide less useful information for the prediction of the onset of PD.

Deposition of p*α*Syn was detected in the ENS of patients with premotor PD. As Wakabayashi et al. reported [13], it is known that Lewy pathology is most commonly detected in the myenteric plexus, with the submucosal layer being the next most common [15], suggesting that detection of p*α*Syn-positive aggregates in the muscularis propria would predict the onset of motor symptoms of PD more sensitively than in the submucosa and mucosa. Several studies have investigated Lewy pathology in the GI tract in the premotor phase of PD [26, 27]. In support of our results, recent meta-analyses have shown that the combined use of anti-p*α*Syn antibody and neuronal markers can increase the sensitivity of Lewy pathology detection [26] and, moreover, that biopsied samples often do not contain the muscularis propria/myenteric plexuses, leading to a decrease in sensitivity [26, 28].

The accumulation of p*α*Syn was not detected in the ENS in four premotor PD patients (P4–P7). This may be because the operation had been performed at a stage that was too early, without patients having any evidence yet of constipation (P4–P6). Previous studies have reported that, within the GI tract, the lower esophagus has the highest frequency of p*α*Syn histopathology, followed by the stomach, while the colon and rectum have the lowest [13, 15, 29]. In contrast, recent reports have revealed that the colon is more sensitive for the detection of Lewy pathology than the stomach in living patients with PD [26, 27]. These observations suggest that we must take sampling site errors into consideration when p*α*Syn is not detected.

The control of GI motility and secretion also depends on both extrinsic parasympathetic and sympathetic innervation [16, 30]. Extrinsic parasympathetic inputs originate in the dorsal motor nucleus of the vagus nerve and in the sacral parasympathetic nucleus, both of which control the motility of the upper GI tract and the distal colon and rectum [31]. The myenteric plexus primarily controls the activity of the smooth muscle of the gut and thus intestinal motility, whereas the submucosal plexus is involved in the regulation of mucosal functions such as secretion and blood flow [32]. It has been reported that dopaminergic defects are seen in the muscularis propria, but not in the mucosa, in PD patients with chronic constipation [33]. Our results suggest that neuronal dysfunction due to the accumulation of p*α*Syn may initially occur in the myenteric plexus, which could lead to alimentary tract dysfunction and induce delayed gastric emptying constipation due to slow peristalsis. Finally, pathological *α*Syn would propagate from the ENS into the brainstem through the vagus nerve, as shown in animal experiments [34].

In the present study, we did not find any relationship between the etiology of gastrointestinal diseases, biopsy sites, and accumulation of p*α*Syn. This was likely because the number of patients was small and all the samples containing muscularis propria originated from the stomach. Moreover, it remains unclear whether the digestive symptoms were induced by gastrointestinal diseases or the premotor symptoms of PD. Further study in a larger population is necessary.

## 5. Conclusion

In conclusion, the deposition of p*α*Syn was observed in the ENS in both the stomach and colon in PD patients prior to the onset of motor symptoms, which could thereby be used as a biomarker for prodromal PD. More importantly, our results suggest that the investigation of GI mucosa and submucosa by immunohistochemistry for *α*Syn might overlook Lewy pathologies and lead to misdiagnoses, but suggest that the detection of p*α*Syn-positive aggregates in the GI muscularis propria could be more sensitive in the prediction of the onset of PD. This study lays the foundation for future research aimed at the development of further useful clinical implementations of these results.

## Figures and Tables

**Table 1:** Clinical profiles of PD patients.

Patient	Sex	Diagnosis/operation	Age at operation	Digestive symptoms^*∗*^	Age at PD onset (initial symptoms)	Biopsy site
P1	M	GC/DG	75	Constipation	75 (dysphagia)	
P2	M	GC/DG	78	Constipation	79 (gait disturbance)	
P3	M	GC/DG	59	Nausea	71 (resting tremor)	
P4	F	CP/EMR	64	Fecal occult blood	73 (resting tremor)	Descending colon
P5	M	CP/EMR	58	Fecal occult blood	60 (resting tremor)	Descending colon
P6	F	GP/EMR	45	Nausea	53 (gait disturbance)	Corpus
P7	F	GP/EMR	52	Constipation	53 (gait disturbance)	Corpus

GC, gastric cancer; DG, distal gastrectomy; CP, colon polyp; GP, gastric polyp; EMR, endoscopic mucosal resection. ^*∗*^Digestive symptoms shown before the operation. The initial motor symptoms of PD are also described.

**Table 2:** Clinical profiles of the control patients.

Patient	Diagnosis	Age	Sex	Age at operation	Digestive symptoms	Biopsy site
C1	Sepsis	74	F	(Autopsy)		
C2	MI	76	M	(Autopsy)		
C3	HCC	75	M	(Autopsy)		
C4	GC	71	M	(Autopsy)		
C5	GC	89^*∗*^	F	80, DG	None	
C6	GC	76^*∗*^	F	70, DG	None	
C7	GC	79^*∗*^	M	72, DG	None	
C8	GC	74^*∗*^	M	67, DG	None	
C9	CC	79^*∗*^	F	73, Sig	Bloody stool	
C10	CC	73^*∗*^	F	67, RH	Bloody stool	
C11	CC	66^*∗*^	M	60, Sig	Fecal occult blood	
C12	CC	62^*∗*^	M	55, Sig	None	
C13	EGC	62^*∗*^	M	55, ESD	None	Corpus
C14	EGC	61^*∗*^	F	56, ESD	Heartburn	Corpus
C15	EGC	68^*∗*^	M	62, ESD	None	Antrum
C16	EGC	67^*∗*^	M	61, ESD	None	Corpus
C17	GP	82^*∗*^	F	76, EMR	Hematemesis	Corpus
C18	CP	79^*∗*^	M	71, EMR	Fecal occult blood	Descending colon
C19	CP	64^*∗*^	F	58, EMR	None	Ascending colon
C20	CP	75^*∗*^	M	69, EMR	None	Ascending colon
C21	CP	69^*∗*^	M	56, EMR	Diarrhea	Descending colon
C22	CP	74^*∗*^	F	67, EMR	Fecal occult blood	Sigmoid colon

MI, myocardial infarction; HCC, hepatocellular carcinoma; GC, gastric cancer; CC, colon cancer; EGC, early gastric cancer; GP, gastric polyp; CP, colonic polyp; DG, distal gastrectomy; Sig, sigmoidectomy; RH, right hemicolectomy; ESD, endoscopic submucosal dissection; EMR, endoscopic mucosal resection. ^*∗*^Age at the latest consultation without neurological symptoms.

**Table 3:** Immunohistochemical findings in patients.

Patient		*α*-Synuclein			Phosphorylated *α*-synuclein		
		MP	SM	Mucosa	MP	SM	Mucosa
C1	Stomach	++	+	−		−	−
	Colon	+	−	−	−	−	−
C2	Stomach	++	+	−	−	−	−
	Colon	++	++	−	−	−	−
C3	Stomach	++	+	−	−	−	−
	Colon	+++	++	−	−	−	−
C4	Colon	++	+	−	−	−	−
C5	Stomach	+++	++	−	−	−	−
C6	Stomach	++	++	−	−	−	−
C7	Stomach	++	+	−	−	−	−
C8	Stomach	++	+	−	−	−	−
C9	Colon	+++	++	−	−	−	−
C10	Colon	+++	++	−	−	−	−
C11	Colon	+++	++	−	−	−	−
C12	Colon	+++	++	−	−	−	−
C13	Stomach	NE	−	−	NE	−	−
C14	Stomach	NE	+	−	NE	−	−
C15	Stomach	NE	−	−	NE	−	−
C16	Stomach	NE	+	−	NE	−	−
C17	Stomach	NE	+	−	NE	−	−
C18	Colon	NE	−	−	NE	−	−
C19	Colon	NE	−	−	NE	−	−
C20	Colon	NE	+	−	NE	−	−
C21	Colon	NE	−	−	NE	−	−
C22	Colon	NE	−	−	NE	−	−
P1	Stomach	+++	++	−	+	−	−
P2	Stomach	+++	+	−	+	−	−
P3	Stomach	+++	+	−	+	+	−
P4	Colon	NE	+	−	NE	−	−
P5	Colon	NE	−	−	NE	−	−
P6	Stomach	NE	−	−	NE	−	−
P7	Stomach	NE	−	−	NE	−	−

Staining pattern: strong (+++), moderate (++), weak (+), and absent (–) for *α*-synuclein and positive (+) or negative (–) for phosphorylated *α*-synuclein. MP, muscularis propria; SM, submucosa; NE, not examined because MP was not included in the tissue samples.

## Data Availability

The data used to support the findings of this study are available from the corresponding author upon request, following approval by the responsible ethical committee.
